# Exploring the potential of mapped soil properties, rhizobium inoculation, and phosphorus supplementation for predicting soybean yield in the savanna areas of Nigeria

**DOI:** 10.3389/fpls.2023.1120826

**Published:** 2023-04-11

**Authors:** Martin Jemo, Krishna Prasad Devkota, Terence Epule Epule, Tarik Chfadi, Rkia Moutiq, Mohamed Hafidi, Francis B. T. Silatsa, Jibrin Mohamed Jibrin

**Affiliations:** ^1^ AgroBiosciences Program, College for Sustainable Agriculture and Environmental Sciences, Mohammed VI Polytechnic University (UM6P), Bengeurir, Morocco; ^2^ Soil, Water, and Agronomy (SWA) Program, International Center for Agricultural Research in the Dry Areas (ICARDA), Rabat-institute, Rabat, Morocco; ^3^ International Water Research Institute (IWRI), College for Sustainable Agriculture and Environmental Sciences, Mohammed VI Polytechnic University (UM6P), Bengeurir, Morocco; ^4^ National Institute of Agronomical Research (INRA), Regional Center of Kenitra, Kenitra, Morocco; ^5^ Cadi Ayad University, Laboratory of Microbial Biotechnologies, Agrosciences and Environment, Faculty of Science Semlalia, Marrakesh, Morocco; ^6^ Center of Excellence for Soil and Fertilizer Research in Africa (CESFRA), College for Sustainable Agriculture and Environmental Sciences, Mohammed VI Polytechnic University (UM6P), Benguerir, Morocco; ^7^ Centre for Dryland Agriculture, Bayero University, Kano, Nigeria

**Keywords:** bradyrhizobium inoculation, foresight IMPACT model, Nigeria savanna agroecologies, participatory on-farm experiment, random forest model

## Abstract

Rapid and accurate soybean yield prediction at an on-farm scale is important for ensuring sustainable yield increases and contributing to food security maintenance in Nigeria. We used multiple approaches to assess the benefits of rhizobium (Rh) inoculation and phosphorus (P) fertilization on soybean yield increase and profitability from large-scale conducted trials in the savanna areas of Nigeria [i.e., the Sudan Savanna (SS), Northern Guinea Savanna (NGS), and Southern Guinea Savanna (SGS)]. Soybean yield results from the established trials managed by farmers with four treatments (i.e., the control without inoculation and P fertilizer, Rh inoculation, P fertilizer, and Rh + P combination treatments) were predicted using mapped soil properties and weather variables in ensemble machine-learning techniques, specifically the conditional inference regression random forest (RF) model. Using the IMPACT model, scenario analyses were employed to simulate long-term adoption impacts on national soybean trade and currency. Our study found that yields of the Rh + P combination were consistently higher than the control in the three agroecological zones. Average yield increases were 128%, 111%, and 162% higher in the Rh + P combination compared to the control treatment in the SS, NGS, and SGS agroecological zones, respectively. The NGS agroecological zone showed a higher yield than SS and SGS. The highest training coefficient of determination (R^2^ = 0.75) for yield prediction was from the NGS dataset, and the lowest coefficient (R^2^ = 0.46) was from the SS samples. The results from the IMPACT model showed a reduction of 10% and 22% for the low (35% adoption scenario) and high (75% adoption scenario) soybean imports from 2029 in Nigeria, respectively. A significant reduction in soybean imports is feasible if the Rh + P inputs are large-scaled implemented at the on-farm field and massively adopted by farmers in Nigeria.

## 1 Introduction

Soybean [*Glycine max* (L.) Merr.] is an important component in smallholder cropping systems due to its rich source of edible proteins, amino acids, and micronutrients, which are indispensable to addressing food insecurity and quality problems (Chigeza et al., 2019; Siamabele, 2021; Alabi et al., 2022). In Africa, soybeans are grown over more than 2.5 million hectares, and Nigeria is the second-largest producer after South Africa (FAOSTAT, 2022). Its cultivation confers several environmental benefits, such as biological nitrogen fixation (BNF) that converts atmospheric nitrogen gas (N_2_) into soil nitrogen (N) for plant uptake (Thilakarathna and Raizada, 2018; Herridge et al., 2022; Ladha et al., 2022). This process contributes to alleviating N deficiencies and improving soil health, soil fertility, and crop productivity (Grönemeyer and Reinhold-Hurek, 2018). In Africa, the promotion of BNF can significantly increase soybean yield, where it is the lowest (only 1.2 t ha^−1^) as compared to the world average (2.8 t ha^−1^), the Americas (3.2 t ha^−1^), Europe (2.0 t ha^−1^), and Asia (1.4 t ha^−1^) (FAOSTAT, 2022).

Seed inoculation with *Bradyrhizobium japonicum* elite strain is a proven strategy to improve soybean yield (Hungria et al., 2017). However, higher biological N fixation and yield response are reported when the legume plants are fertilized with a moderate phosphorus (P) rate, particularly in many soils and climatic conditions in Africa where available P in the soil is low (Jemo et al., 2010). The use of an appropriate strain of Rh inoculant and P fertilization practices to improve BNF legume production has been the subject of numerous studies in Sub-Saharan Africa (Ronner et al., 2016; Ulzen et al., 2018; van Heerwaarden et al., 2018; Buenor et al., 2022). Those studies have reported yield increases ranging from 452 to 815 kg ha^−1^ and a net economic benefit of about 400 USD ha^−1^ through the combined application of Rh inoculants and P fertilizer. Despite the above-mentioned advantages from the combined application of Rh inoculants and P supplementation to soybeans, there are obstacles to achieving higher yields due to the divergent impacts of many abiotic and biotic factors like drought, nutrient availability, and crop genotypes (Alves et al., 2003; Jemo et al., 2006; Ulzen et al., 2018; Khaki et al., 2020).

In Africa, the import of soybean products has witnessed an exponential increase, with more than 14 million USD spent on soybean imports in 2020 alone for Nigeria (FAOSTAT, 2022). As a consequence, the country is highly dependent on international soybean trade, which places pressure on household resources and negatively impacts food security and nutrition. Therefore, it is imperative to increase the yield of the crop per hectare of land to meet national and regional food demands with minimal environmental damage (Helfenstein et al., 2020). Yield prediction is complex, but accurate prediction provides timely import and export decisions to policymakers and provides year-to-year management and financial decisions to farmers (Smidt et al., 2016; Khaki and Wang, 2019). Yield prediction of crops, including soybeans, has been the subject of studies, but the prediction results, in general, are often challenging due to the interactions among numerous complex factors such as crop genetics, weather, soil input and crop management, and socio-economic conditions (Smidt et al., 2016; Khaki et al., 2020; Alabi et al., 2022; Bebeley et al., 2022). When using soil properties to predict soybean yield, soil available P, organic matter, soil available water supply in the upper 100 cm, and soil K were the major yield determinants (Smidt et al., 2016). In sub-Saharan Africa, using multispectral high-resolution unmanned aerial vehicles, Alabi et al. (2022) estimated soybean grain yield in on-station trials, focusing on varietal evaluation approaches and rapid high throughput phenotypic workflows. Another recent modeling study evaluated the CROPGRO−soybean model for assessing optimum sowing windows of soybean in the Nigeria Savannas and found that sowing dates between 15 June and 5 July accurately predicted the yields of genotypes TGX1951−3F and TGX1835−10E (Bebeley et al., 2022). However, limited studies have accounted for integrated soil properties, weather, and crop management practices for soybean yield prediction across Nigerian agroecology. Public availability of prediction datasets, the associated high costs, time consumption for analyses, and the sample size curtail acute prediction in such studies (Hengl et al., 2021; Wang et al., 2022). Thanks to a recent Soil Information System for Africa (iSDAsoil) mapped at 30 m resolution that is now making it possible to integrate them into models for predictions of African crops. The iSDAsoil platform provides detailed pan-african soil macro and mincronutrients maps at fine spatial resolutions (Hengl et al., 2021). However, for crops like soybeans, an important food security crop that has rapidly expanded in Africa, yield prediction is yet to be implemented.

Ensemble learning, which is a combination of several machine learning models, has made it feasible to combine several factors for predicting yields with robust results. Various techniques of ensemble learning, such as regression, decision trees, association rule mining, artificial neural networks, and random forest (RF) models, provide results by combining several base models and datasets. Multivariate regression and random forest machine learning approaches have been recently applied to crop yield prediction (Khaki and Wang, 2019; Khaki et al., 2020). A salient feature of machine learning models is the holistic assessment of the input variables, which are often non-linear and complex functions of the output variable, such as crop yield (Khaki and Wang, 2019; Khaki et al., 2020). Machine learning techniques such as RF regression have been previously used to quantify the predictors of importance to outputs and identify the optimal input ranges as an entry point for closing the yield gap sustainably (Breiman, 2001; Devkota et al., 2021).

Furthermore, global food security is challenged by rapid changes in population, income, and climate change. Achieving and maintaining these threats and designing possible solutions requires a robust multidisciplinary approach (Robinson et al., 2015; Islam et al., 2016). The International Model for Policy of Agricultural Commodities Trade (IMPACT) model was developed by the International Food Policy Research Institute (IFPRI) links economic, water, and crop modules to simulate domestic and international agricultural markets and support needs under changing biophysical and socio-economic conditions and provides in-depth analysis and decision-making support to policymakers (Mason-D’Croz et al., 2016).

The adoption of Rh + P fertilizer technology can sustainably increase soybean yield per hectare at the national and regional scales, aid in reducing dependency on the international market for soybean products and contribute to food security maintenance. Therefore, the objectives of the present study were to: (a) examine the soybean yield variation as affected by Rh + P application across three agroecological zones of northern Nigeria; (b) predict soybean yield change using digitalized soil properties data and machine learning techniques; and (c) explore the scenarios of adoption of the combination Rh + P impacts on soybeans, reducing imports by 2050.

## 2 Materials and methods

### 2.1 Experimental site

**Figure 1:**
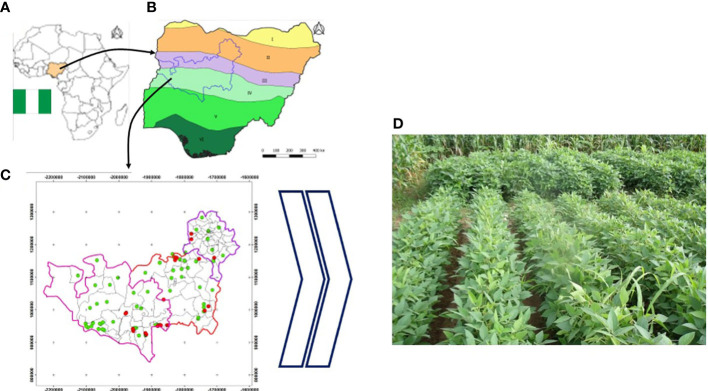
Map of Africa **(A)** and the different agroecological zones in Nigeria **(B)**, on-farm demonstrations areas **(C)**, and different field operations **(D)** field Rhizobia + P fertilizer combination and non-inoculated plots.

On-farm demonstration trials were conducted for two years (2012–2013) in three agroecological zones of northern Nigeria (9° 05’ to 11° 54’ N and from 6° 38’ to 8° 17’ E), particularly covering the Sudan Savanna (SS), northern Guinea Savanna (NGS), and southern Guinea Savanna (SGS) regions (**Figure 1**). Long-term rainfall ranges from 600 to 1,000 mm (mean of 744.5 mm) in the SS, from 1,000 to 1,300 mm (mean of 1,179 mm) in the NGS, and from 1,100 to 1,400 mm (mean of 1,270 m) in the SGS (Ishaku and Majid, 2010; Umar and Bako, 2019). The extracted cumulative precipitation, average minimum, and maximum temperature are reported in **Table 1**.

**Table 1:** Minimal (min), median, and maximum (max) of cumulative monthly precipitation (mm), average of minimal and maximal temperature, and covered administrative local governments of studies in the Sudan, Northern Guinea, and Southern Guinea Savannas of Nigeria recorded for 2012 and 2013 growing seasons.

Agroecological zones		Cumulative annual precipitation (mm)		Average minimum temperature (°C)^1^		Average maximum temperature (°C)		Covered administrative local governments
		2012	2013	2012	2013	2012	2013	
Sudan Savanna	Min	320.2	358.6	16.6	16.8	35.2	35.6	DANJA, GEZAWA, GWARZO, KARA, KARAYE, KURA, RANO, SOBA, and UNGOGO
	Median	390.3	413.6	18.0	19.5	37.9	37.5	
	Max	420.0	580.1	19.0	20.4	39.1	39.1	
Northern Guinea Savanna	Min	569.6	437.7	16.3	16.3	33.7	34.1	GIWA, IGABI, JEMA’A, KUDAN, Sabon GARI, and Zango KATAF
	Median	659.8	597.4	17.1	16.9	35.5	35.4	
	Max	712.5	754.1	18.0	18.0	35.8	36.1	
Southern Guinea savanna	Min	390.3	580.1	16.6	16.5	32.9	33.5	ABAJI, AGAIE, BOSSO, GURARA, IGABI, KATCHA, MUYA, SHIRORO, SULEJA, TUFA, and WUSHISHI
	Median	1252.1	680.6	18.5	18.0	33.9	33.8	
	Max	1629.5	717.2	19.7	20.7	37.9	35.5	

### 2.2 Experimental design, treatments, and crop management practices

A total of 350 on-farm demonstration experiments were conducted across three agroecological zones in Nigeria. Those on-farm experiments were conducted using a randomized complete block design (RCBD), considering each farmer’s field as a replicate. Each plot measured 6 × 4 m^2^, and 350 experimental fields were established in the three agroecological zones. The seeds of a soybean variety were hand-drilled at 2 cm depth at 0.75 m between rows and 0.05 m within rows, recovered with soil, and thinned to 5 cm distance between plants after 15 days to maintain a uniform population density of 266,667 plants per hectare. Four treatments (Trt) were evaluated: Trt 1: Control (farmer practice) without inoculation and no P application (non-treated); Trt 2: P-fertilizer application at 20 kg P ha^−1^ (P) in the form of triple superphosphate (TSP) (Ronner et al., 2016); Trt 3: Rh inoculation at 5 g kg^−1^ seed; and Trt 4: Combined application of Rh and P. Soybean seeds were treated with a commercial Rh inoculant (legume-fix) containing 10^10^ bacteria cells of *B. japonicum* strain 532c per gram of solid before sowing. The Rh inoculant was coated onto the seeds with gum arabic as a sticker and air-dried for 30 min under shade before sowing. The P fertilizer was applied by hand-broadcast within rows at sowing. The trained extensionists and farmers managed the experimental plots subsequently. Weed management was carried out through regular hand weeding every 30 days at intervals in consultation with the extensionist. Farmer groups and rural community members regularly visited the experimental plots during field days organized at the vegetative growth stage to demonstrate the treatment differences with the support of Village Promoter Agents (VPAs).

### 2.3 Pre-campaign training of village promoter agents and farmers groups for on-farm experimentation

Village promoter agents (VPAs) were recruited by the area manager staff of the Notore Limited group in Nigeria to monitor the trials. The VPAs were local farmers based in the villages with previous working experience in monitoring demonstration trials, good communication skills in Hausa local language with farmers, and an interest in participatory technology dissemination to rural farmers. Notore Limited is an established private sector company based in Nigeria with a recruited area manager who daily supervises the work of VPAs in the deployed areas. Each VPA received adequate training at the early stages before the on-farm demonstration establishment of trials regarding the handling of rhizobial inoculants, coating seeds, and P applications in the respective areas. Twenty-five (25) VPAs were trained for handling rhizobial inoculants, P fertilizer applications, and general monitoring of the trials. Collaborative farmer groups and community contact persons for participatory research were registered and trained. Identified fields to establish the demonstration trials were delimited, and the geographical coordinates of each field were recorded (**Figure 1**). Thereafter, farmer groups were subsequently trained to handle rhizobium inoculant for seed coating techniques in the respective locations, ensuring limited risks of cross-contamination. The sowing order was control, P treated, Rh inoculated, and Rh + P fertilizer plots, respectively. Farmers’ groups and VPA regularly visited the demonstration plots at various growth stages, from sowing to harvest.

### 2.4 Soybean varieties and rhizobium inoculant

Three improved soybean varieties of different maturity groups developed by the International Institute of Tropical Agriculture (IITA) in Nigeria and released by the Nigeria National Research System (https://www.seedportal.org.ng) were used for the trials. The varieties were derivatives of a tropical *G. max* (TGx) series of cultivars bred for their promiscuous nodulation in a wide range of environments. The soybean varieties TGx 1987-62F (reg.: NGGM 10-19) and TGx 1987-10F (reg. NGGM 10-18) were released in 2010 and are resistant to Cercospora leaf spot and bacteria pustules. The TGx 1987-62F variety is a medium maturity group (90–110 days to maturity) and was used in demonstration plots in NGS agroecology. This variety had an average grain yield of 2.1 t ha^−1^ in on-station rainfed trials in Nigeria (https://www.seedportal.org.ng). The soybean variety TGx 1987-10F is also highly resistant to Cercospora leaf spot and bacterial pustules, with a yield range of 1.5–2 t ha^−1^ under rainfed conditions. It is an early maturity variety (90–95 days to maturity) and was used in the experimental plots in the SS agroecology. The third variety, TGx 1448-2E, was released in 1992 and registered in 1996 under the Nigerian national code NGGM-96-15. It has an average grain yield of 2.4 t ha^−1^, is frog-leaf resistant and belongs to the late maturity group (115–120 days); this variety was used in the SGS agroecology.

### 2.5 Data acquisition, preparation, and random forest and IMPACT models implementation

#### 2.5.1 Grain yield

The soybean plants were harvested at maturity 90 to 110 days after sowing. Dried plants were harvested from the entire plot (24 m^2^). Grains were separated from pods and sun-dried, and the dry weight of the seeds was recorded. The grain yield expressed in kg ha^−1^ was computed using Equation 1 (Eq. 1).


$$Yield\;\left(kg/ha\right)\;=\;[(Net\;plot\;yield\;\left(g\right)\;∕\;1,000\;\left(g\right)\;\times \;((Area\;\left(ha\right)\;10,000\;\left(m^{2}\right)/Net\;plot\;area\;\left(m^{2}\right)\;\times \;((100-MC)/88)]$$

where MC is moisture content (%). (Eq. 1) (Awuni et al., 2020).

#### 2.5.1 Soil properties and weather data for predicting yield

Soil properties for each experimental site at 30 m spatial resolution were extracted from the iSDAsoil (https://www.isda-africa.com/isdasoil/) platforms using the “raster,” “rgeos,” “maptools,” “rgdal,” “shapefiles,” and “PBS mapping” functions of the R packages (R version 4.2.1). The minimum median and maximum values of the extracted soil properties are given in **Table 2**. Specifically, soil pH, organic carbon (C), and total nitrogen (N), total carbon, effective cation exchange capacity (ECEC), available phosphorus (P), exchangeable potassium (K), exchangeable calcium (Ca), exchangeable magnesium (Mg), sulfur (S), sodium (Na), iron (Fe), zinc (Zn), silt, clay, and sand variables were extracted for each experimental site (**Table 2**). Monthly precipitation, temperature, and solar radiation for each site during the crop-growing season were extracted from the NASA platform (https://power.larc.nasa.gov/data-access-viewer/).

**Table 2:** Maximum (Max), median and minimum (Min) of soil properties from all sampled sites, Sudan, northern Guinea, and southern Guinea Savannas of Nigeria.

	All sites			Sudan Savanna			Northern Guinea Savanna			Southern Guinea Savanna		
	Min	Median	Max	Min	Median	Max	Min	Median	Max	Min	Median	Max
Effective Cation Exchange Capacity [cmol (+) kg^−1^]	7.4	12.2	16.4	9.0	13.5	16.4	7.4	13.5	14.9	7.4	9.0	16.4
Exchangeable Ca [cmol (+) kg^−1^]	0.90	3.0	7.3	2.7	2.7	6.02	2.0	4.5	7.3	1.0	3.6	5.4
Fe content (mg kg^−1^)	27.1	33.1	54.6	27.1	27.1	40.4	27.1	32.7	40.4	30.0	3.6	54.6
Exchangeable Mg [cmol (+) kg^−1^]	0.49	1.0	2.0	0.66	0.99	1.34	0.60	0.90	2.00	0.45	0.98	1.64
Av-Pi content (mg P kg^−1^)	6.0	7.4	10.0	6.0	6.6	10.	6.0	7.4	9.0	6.0	7.4	10.0
Exchangeable K [cmol (+) kg^−1^]	0.48	0.6	0.74	0.49	0.66	0.74	0.49	0.60	0.74	0.45	0.54	0.67
Su content (mg kg^−1^)	3.7	4.95	6.7	3.7	4.9	6.0	4.1	5.0	5.0	3.7	4.5	6.7
Zn content (mg kg^−1^)	1.5	2.2	4.1	1.5	2.2	2.7	1.5	2.4	3.3	1.5	1.8	4.1
Organic carbon content (g kg^−1^)	4.1	5.5	13.5	4.1	5.5	10.0	4.1	4.9	10.0	4.5	4.9	13.5
Total Nitrogen content (g kg^−1^)	1.3	1.7	2.2	1.4	1.6	2.2	1.3	1.7	2.1	1.4	1.7	2.2
pH (H_2_O)	5.1	5.7	6.1	5.5	5.7	6.0	5.3	5.7	6.1	5.1	5.6	5.8
Clay content (%)	16.0	24.0	32.0	18.0	22.6	26.0	16.0	25	30.0	19.0	23	32.0
Silt content (%)	43.0	54.0	67.	45.0	54.0	60.0	43.0	52	67.0	46.0	56	61.0
Sand content (%)	15.0	20.0	26.0	18.0	23.4	24.0	18.0	22.4	26.0	15.0	18	23.0

### 2.6 Random forest machine learning for yield prediction

**Figure 2:**
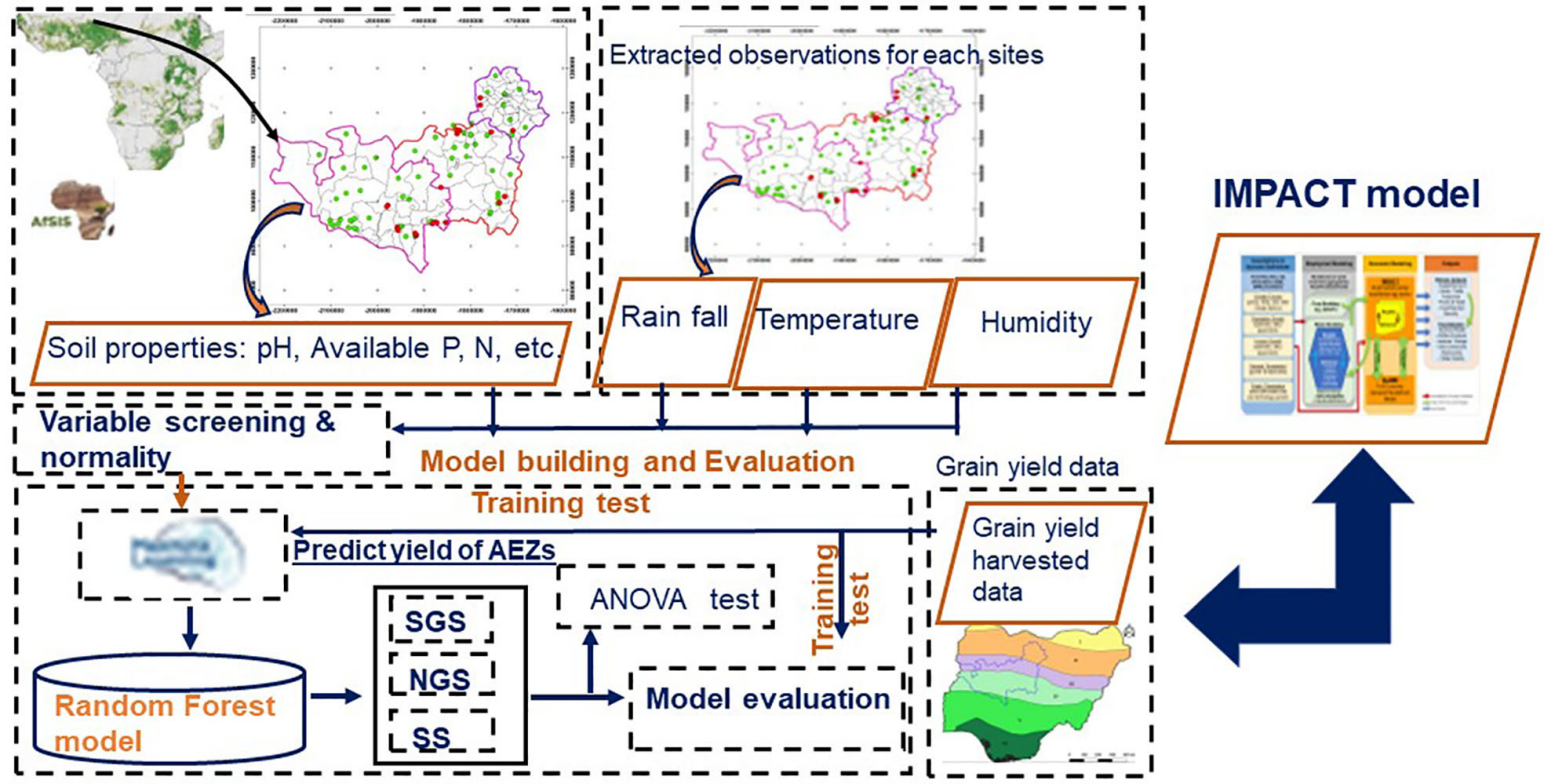
Framework of the model implementation approach. ANOVA, Analysis of variance; SGS, southern Guinea Savanna; NGS, northen Guinea Savanna; SS, Sudan Savanna; AEZs, Agroecological zones.


A logical framework for the model’s implementation, calibration, and training is displayed in **Figure 2**. A conditional inference regression RF machine learning approach was implemented for predicting yield variability from each agroecological zone. The conditional RF captures the linear and non-linear effects of the estimator variables (soil, weather, and factor variables) on the yield response and quantifies the marginal effect of individual inputs. The inference regression RF is a powerful non-parametric decision ensemble learning method for regression classification that operates by constructing multiple artificial trees to predict and fit response variables without overfitting during the training process. To assess the model’s performance, the root mean square error (RMSE), training coefficient of determination (R^2^), and validation RMSE were computed for the datasets of each agroecoregion. The predicted values against the actual and variables of importance for each model were visualized. The normalized RMSE (NRMSE) was calculated using the formula:

$$NRMSE\;\left(\%\right)\;=\;[RMSE/(y_{max}-y_{min})]\;\times 100$$

(Eq. 2, Alabi et al., 2022),

where y_max_ and y_min_ are the maximum and minimum yield.

#### 2.6.1 Screening variables of importance for the better prediction

Variables with low importance were discriminated from the principal component analysis check to reduce dimensionality in the number of input variables in the training dataset. A forward selection of explanatory variables for yield was performed, and predicted variables with a *P*-value below<0.05 were retained and included in the training dataset. Twenty-eight estimators from a total of 66 variables aggregated as predictors were retained for yield prediction of datasets from all agroecological zones, whereas 26 estimators were screened for model training for each agroecological zone dataset.

#### 2.6.2 Training, testing, and model fitting

The training dataset was built with 70% of the dataset (by dividing the 1,400 observations by 0.7) and tested on 30% of the remaining dataset in the R package (version 4.2.1). The “cforest” functions of the partykit package in R were used for the model fit using unconditional subclasses, 200 as a number of trees, and 5 input variables randomly sampled as candidates at each node.

### 2.7 Application of IMPACT model

#### 2.7.1 Model framework

The foresight IMPACT model (https://www.ifpri.org/project/ifpri-impact-model) was used to explore soybean marketing and import scenarios during 2017–2050 through the adoption of rhizobium inoculation and supplemental application of P fertilizer. The IMPACT model framework considers components of climate models (Earth System Models), crop models (Decision Support System for Agrotechnology Transfer, DSSAT), water models (hydrology, water basin management, and water stress models), land-use models (pixel-level land use) and integrates them into the multi-market model. The IMPACT model computes the effects of national and international demand and prices and is designed for scenario analysis rather than forecasting (Robinson et al., 2015).

#### 2.7.2 Model integration, model inputs, and scenario analysis

In the IMPACT model, crop yield is a function of commodity price, input prices, available water, climate, and market variables. The model integrates five modules (climate, crop, water, land use, and market) to assess changes in yields. The model assumes a scenario of underlying improvements in yields due to the adoption of technology and simulates crop yields in specific areas as functions of the introduction of technology (Eq. 2).


$$Yield_{it}=\;\sum \left(Soy\_adtech_{it}\times Soytech\_Yield_{it}\right)$$

Eq. 2. (Robinson et al., 2015)

Where, *Soy_adtech* = Soybean adoption technology for a country *i* at the period *t*, and *Soy_tech =* Soybean inoculation technology for a country i at the period *t* and under no climate change effect.

Two future scenarios were assessed in this study:

- a moderate adoption scenario where the adoption rate of the Rh + P fertilizer combination among farmers stops at 35%, and
- a more extensive scenario in which the adoption rate of the Rh + P fertilizer combination reaches 75%.

For both scenarios, it was assumed that the adoption of the Rh + P fertilizer technology would happen gradually between 2017 (the first year of beginning adoption) and 2050 (the year in which the model is calibrated for inputs). The effect of improved soybean inoculation technology and P fertilization was simulated by reducing imports and saving currency in Nigeria.

### 2.8 Statistical analysis

General statistical analysis was carried out for the three agroecological zones), and yield prediction using machine learning with 68 constructed explanatory variables and a single yield response variable was carried out. The extracted soil properties used to predict yield were tested for normality, skewness, and the kurtosis test, which reported a P value of<0.05. Descriptive statistics (maximum, median, and minimum) were computed for the yield variable. A one-way analysis of variance (ANOVA) was carried out to assess the effect of treatment on grain yield change using the JMP statistical software (JMP, 2019). The treatment mean differences were analyzed using the least significant difference (LSD) at 5% and 1% of the level of significance when the Fischer (F) value was significant from the ANOVA (P<0.05). Levels of significance are given by “ns” (not significant, *P >*0.05), *P<0.05, **P<0.01, and ***P<0.001. The RF analysis was computed using R Studio version 2022.12.0 (R version 4.2.1).

## 3 Results

### 3.1 Soil properties

Descriptive statistics of extracted soil properties used for model training and validation are presented in **Table 2**. Averaged across the agroecological zones, the available P ranged from 6.0 to 10.0 mg kg^−1^ and from 6 to 9 mg kg^−1^ in the NGS, with a right skew data distribution range (**Table 2** and **Table S1**). Similarly, for the three agroecological zones, the pH ranged from 5.1 to 6.1, 5.5–6.0 in SS, 5.3–6.1 in NGS, and 5.1–5.8 in SGS, with a left (negative) skew data distribution (**Table S1**). The range of clay contents varied from 16% to 32% for all agroecological zones: 18%–30% in SS, 16%–30% in NGS, and 19%–32% in SGS. Similarly, the silt contents ranged from 43% to 67% across the three agroecological zones: 45%–60% in the SS, 43%–67% in the NGS, and 46%–61% in the SGS agroecology. Other extracted soil property summary statistics, such as their skewness and kurtosis values are reported in **Table 2** and **Table S1**.

### 3.2 Soybean yield response as affected by Rh inoculation and P application

**Figure 3:**
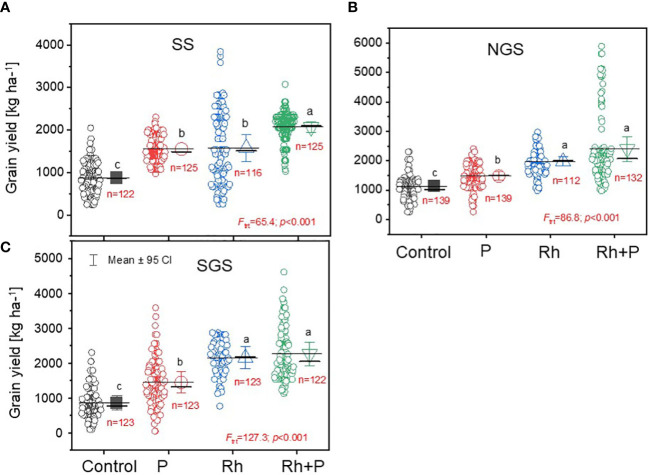
Boxplots of the soybean yield (kg ha^−1^) for the control, phosphorus (P) fertilizer, rhizobia (Rh) inoculants, and the Rh + P combination in the **(A)** Sudan Savanna (SS), **(B)** Northern Guinea Savanna (NGS), and **(C)** Southern Guinea Savanna (SGS) of agroecology of Nigeria. Mean and ±95% confidence intervals are presented. Mean values appended by a different letter indicate significant differences at *P<*0.05.

A one-way ANOVA testing the effect of the treatment on grain yield was highly significant for the SS (F = 65.4, *P<*0.001), NGS (F = 86.6, *P<*0.001), and SGS (F = 127.3, *P<*0.001) agroecological zones, respectively (**Figure 3**). The yield data for the combined application of Rh + P fertilizer was normally distributed in the SS, right-skewed in the NGS, and left-skewed in the SGS agroecological zones (**Figures 3A–C**). The soybean yield of the Rh + P fertilizer treatment was always higher than that of the control treatment in the three agroecological zones of northern Nigeria (**Figure 3**). Average across all on-farm demonstration yield increments of 128%, 111%, and 162% were observed under the Rh + P combination compared to the control in SS, NGS, and SGS, respectively, and the overall increment for all agroecological zones of the established demonstration trial was 134% (**Figures 3A–C**). The average grain yield for the control treatment was the lowest in SGS compared to the SS and NGS agroecologies (**Figures 3A–C**). When inoculated with Rh alone, soybean yield was always higher in the Rh treatment than in the control treatment in the respective agroecological zones (**Figures 3A–C**). Similarly, the yield of the P fertilized treatment was higher than the control in the SS, NGS, and SGS agroecological zones, respectively (**Figures 3A–C**).

### 3.3 Soybean yield prediction using random forest machine learning

**Figure 4:**
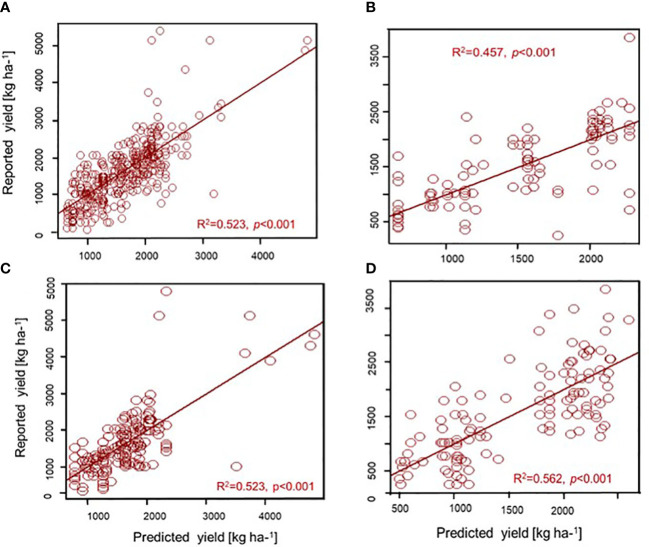
Scatter plots of the soybean predicted and reported yields validation from all sample sets **(A)**, Sudan **(B)**, Northern Guinea **(C)**, and Southern Guinea **(D)** Savannas of Nigeria. The validated coefficient of determination (R^2^) of each sample set is indicated.

The results from RF models of data from the three agroecological zones and separately from each agroecological zone are presented in **Table 3** and **Figure 4** with their NRMSE and R^2^ values. Among the three agroecological zones, NGS provided the highest trained R^2^ value of 0.74 (**Table 3**). The trained NRMSE for NGS samples was 8.8 (**Table 3**). The validated R^2^ and NRMSE were 0.52 and 6.0 (**Table 3**). For the SGS, the trained R^2^ was the lowest (0.58) and the trained NRMSE was the highest (12.7) compared to the SS, NGS, and overall samples (**Table 3**). The validated R^2^ and NRMSE were 0.53 and 8.9, respectively (**Table 3**). For the overall dataset, we found trained RMSE and R^2^ values of 8.8 and 0.64, while the validated NRMSE and R^2^ were 6.2 and 0.57, respectively (**Table 3**). The highest trained NRMSE was observed in SGS samples, and the reported R^2^ was 0.58 (**Table 3**). The validated NRMSE and R^2^ for SGS samples were 9.9 and 0.56 (**Table 3**). The biplots of the predicted and observed samples showed more dense points in the overall datasets and the NGS samples (**Figures 4A, C**).

**Table 3:** Training normalized root mean square error (NRMSE) and coefficient of determination (R^2^), validated NRMSE, validated R^2^, sample size, and number used estimators that predicting yield response of soybean from all sample sets, Sudan, Northern Guinea, and Southern Guinea Savannas of Nigeria.

Dataset	Training normalized root mean square error (NRMSE)	Training coefficient of determination (R^2^)	Normalized validation (NRMSE	Validation coefficient of determination (R^2^)	Sample size		Number of estimators
					Training	Tested	
All agroecological zones	8.8	0.64	6.2	0.57	980	420	28
Sudan Savanna	10.1	0.46	8.9	0.53	258	110	26
Northern Guinea Savanna	8.0	0.75	6.0	0.52	419	179	26
Southern Guinea Savanna	12.7	0.58	9.9	0.56	304	130.2	26

**Figure 5:**
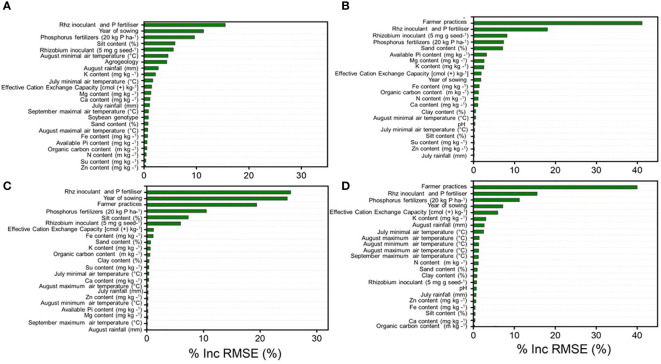
Best inputs variable of importance from soil, weather, and factorial estimators used to predict soybean yields from all sample sets **(A)**, Sudan **(B)**, Northern Guinea **(C)**, and Southern Guinea **(D)** Savannas of Nigeria.

The input variables of importance to predicting yield in the three agroecological zones are presented in **Figure 5**. For the three agroecological zones, the top five variables (based on importance) for predicting yield include the combined application of Rh + P fertilizer, year-to-year growing conditions, silt content in the soil, rhizobium inoculation, and the minimal temperature in the month of August (**Figure 5A**). The top five predicting variables of importance to soybean yield in the Sudan Savanna were crop management practices, combined application of Rh and P fertilizer, rhizobium inoculation, sand content in the soil, and soil available P (**Figure 5B**). The top six predictor variables for yield in the NGS were Rh + P combination, P fertilizer, year-to-year soybean cultivation, crop management practices, P fertilizers, and silt content in the soil (**Figure 5C**). Similarly, the RF model found crop management practices, Rh + P combination, P fertilizer, year-to-year cultivation, and effective cation exchange capacity as the top five yield predictor variables in SS (**Figure 5D**).

### 3.4 Food security and import through the adoption of rhizobium and P fertilizers

**Figure 6:**
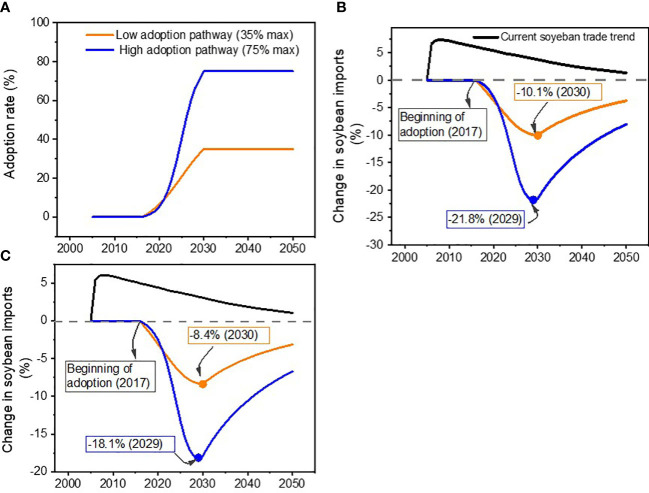
Scenario outlooks (2010–2050) of adoption of the combined rhizobia and phosphorus fertilizers technology based on average yield increase by 133.7% from the Nigeria savannas and by 111% in the Northern Guinea Savanna on-farm demonstrations plots **(A)** adoption profile by 35% and by 75% with with projectd impact under scenario, **(B)** for all sample sets and **(C)** Northern Guinea Savanna.

The simulation using the IMPACT model showed that soybean yield increases through the combined application of Rh and P fertilizer will reduce national trade through the less imports and result in currency savings in Nigeria by 2050. We implemented the model using an average yield increase of 134% (all agroecological zones) and 111% in the NGS and two scenarios of adoption rates: low (35% adoption rate) and high (75% adoption rate) (**Figure 6A**). The model was implemented using the dataset from the NGS agroecological zone because it had the highest prediction and accuracy from the RF model. Considering the average yield increase performance of 134% (averaged across three agroecological zones), results from the IMPACT model scenario showed that the quantity of soybeans imported in the country can be reduced by −10% (35% maximum) and by −22% (75% maximum adoption scenario) if the combined application of Rh and P fertilizer technology is adopted (**Figure 6B**). With an average yield increase of 111% from the combined application of Rh + P (observed in NGS), importation can be reduced by 8.4% (under a low adoption scenario) and by 18% (under a high adoption scenario) by 2030 in the country (**Figure 6C**).

## 4 Discussion

Agricultural technologies focusing on increasing productivity, improving farmers’ profitability, and enhancing sustainability are urgently needed to enhance the household food security of smallholders, particularly in SSA countries (Helfenstein et al., 2020). Such technologies are to be market-oriented, affordable, adapted to smallholder needs, and help to bridge gaps by integrating proper delivery mechanisms. This study demonstrated that on-farm improved soybean rhizobia inoculation technologies tested in collaboration with extension agents can help improve yield and profit, reduce soybean imports, and contribute to food security maintenance in Nigeria. Our results, in accordance with previous studies (Ronner et al., 2016), also demonstrated that yield increments from the combined application of Rh + P fertilizer were always higher than the control (farmer practice) in all three areas of Nigeria. Soybean yield at NGS sites was well predicted by the RF compared to the SS and SGS agroecological zones. A significant reduction in soybean imports in Nigeria could be made through yield increments from the combined application of Rh inoculant and P fertilizer. However, a rapid implementation strategy and massive adoption by farmers are required.

### 4.1 Yield response of soybean as affected by rhizobium inoculation and P application

A series of on-farm demonstration experiments showed soybean yield increased through the combined application of Rh + P (always higher in under-treated conditions than in non-treated conditions), irrespective of the agroecological zones and soil types. In the absence of Rh inoculation, P fertilizer, or Rh + P combination, the average yield in control was 1,084 kg ha^−1^ in the NGS sites, while the yield increased in the Rh + P inoculated plants by 2.3-, 2.1-, and 2.6-fold as compared to the non-treated plants (**Figure 1**). Earlier work demonstrated increased soybean yields with the combination of Rh inoculants and P fertilizer in West African soils. The observed higher yields of 1,188 kg ha^−1^ in SS, 1,203 kg ha^−1^ in NGS, and 1,397 kg ha^−1^ in SGS for the Rh + P application in West Africa (Ronner et al., 2016; Ulzen et al., 2018; Buenor et al., 2022). The authors reported an average yield increment of 815 kg ha^−1^ from the combined application of Rh + P, along with an increment in farmers’ net profit. Several factors accounted for the higher yield under the Rh + P application, such as regular field monitoring by Notore extension agents, high-performing rhizobia microbes, and careful crop management by the engaged farmers during the implementation of the project activities. It is indicated that education, research, and extension in agriculture remain the vehicles to achieve sustainability in the modern food system. The NGS agroecological zone showed a favorable niche for rapidly increase of soybean yield using appropriate management interventions for food security in Nigeria and the sub-region. This high yield can be explained by suitable rainfall and soil fertility conditions. Suitable conditions for optimal soybean production require about 1,000 mm of water in rainfall-based production systems. The SS agroecological zone is a more drought-prone area that often limits yield. The SGS agroecology is the domain of acidic soils and low-P in Nigeria, which are limiting conditions to a high soybean yield (Jemo et al., 2015).

Farmer-managed participatory interventions and extension agents’ engagement certainly facilitated the timely establishment of on-farm demonstrations and weeding at critical crop developmental stages, *inter alia*. Thus, investments in information/knowledge dissemination, input fertilizers, and other technologies are crucial for the sustainable intensification of SSA.

Rhizobia inoculants are perishable commercial products, and this project secured standard and high-quality Rh inoculum with stable self-life from Legume Tech, UK. The inoculant was formulated with bacterial cell concentrations above 10^10^ cells g^−1^ with lyophilized *B. japonicum* that was kept in the Notore stores and delivered to VPAs only a few days before soybean sowing. It is worth mentioning policy decisions aiming to accelerate the manufacturing units, the marketing of high-quality rhizobia inoculants with a satisfying minimum of bacteria cell concentration of 10^9^ cells g^−1^, and the longer shelf-life of rhizobium to accelerate soybean production in Africa, as shown in the success study developed in Brazil (Bomfim et al., 2021). Other incentive measures to increase soybean production in SSA are the institutionalization of P fertilizers and their dissemination to rapidly address pressing food security issues.

### 4.2 Soybean yield prediction using random forest machine learning

Accurate yield prediction is of great importance to global food production. Using digitally soil-mapped properties and extracted weather and management variables, we predicted yield for the three agroecological zones using the RF machine-learning algorithm tools. The highest training R^2^ (0.74) was achieved using samples from the NGS sites (**Table 3**). Alabi et al. (2022) predicted soybean yield using the vegetation index and soil texture information in the RF model. We lacked a comparable study on predicting soybean yield using soil properties in Nigerian Savanna conditions. The results of this study showed that NGS is the predominant agroecology for soybean production in terms of the required soil properties for growth. A better yield prediction in NGS can be explained by symmetrical data distribution (−0.42–0.58) of the extracted soil properties in the NGS agroecological (**Table S1**). The implications are that the soil and climatic conditions of NGS are favored for better growth and less drought effect during the growth stage. Bebeley et al. (2022) evaluated long−term seasonal analysis of soybean yield among the same three agroecological zones for deriving optimal sowing times for different soybean varieties, where yields were simulated in the NGS sites compared to other agroecological zones. In this study, the low R^2^ values of the training dataset from SS and SGS agroecological zones could be attributed to normal data distributions of yield variables, making the yield data from SS and SGS less reliable for their good prediction using soil properties and extracted variables.

Predictions of soybean yield using datasets from the three agroecological zones (i.e., SS, NGS, and SGS) reported that the Rh + P combination has the topmost importance to increase yield under challenging environmental conditions. Also, P-fertilizer and Rh inoculation alone were also among the top variables in importance, but their relative importance varied depending on the agroecology. The available P of the soils was revealed as an important variable for soybean yield in the SS. The results imply that the supply of P fertilizers is largely required if farmers are to grow soybeans in the SS, SGS, and NGS soils. Other important soil properties, such as silt and sand contents and ECEC, were also predictors of soybean yield (**Figure 5**). The present study extracted an average silt content of 53.3% and a sand content of 19.9% (**Table 2**). Apart from precipitation, temperature, and macro- and micronutrients, crop yields are also dependent on soil properties such as soil texture that influence water retention at the root zones and improve nutrient diffusion and crop yield (Huang et al., 2021). Fine-textured or silty loam soil provides a higher water holding capacity and more resistance to plant water uptake in wet conditions compared to sandy soils and can be poorly drained and susceptible to waterlogging, which can lead to denitrification and yield loss (Huang et al., 2021). The interactions between physical soil properties and soybean yields are not well quantified across the agroecological zones of Nigeria and deserve further research and investigation. Such information is necessary to design key indicators to improve soil structure and carbon stocks to increase soil availability for water storage and nutrient retention and promote energy conservation around the soybean root zones.

The minimum air temperature recorded in August was among the top five predictors of importance to soybean yield. These temperatures correlated with the soybean pod filling stages and were in the range of 17–20°C, which was above the air temperature (15°C) reported to inhibit seed filling. The optimum temperatures for soybean are 15–22°C at the emergence stage, 20–25°C at the flowering stage, and 15–22°C at the maturity stage, and seed yield and yield formation of soybean are frequently reduced by temperatures below 15°C and above 30°C (Zhang et al., 2016).

In the present study, we observed that the R^2^ for actual and predicted yields was less than 60% (**Table 3** and **Figure 3**). The results imply that all the aggregated soil and weather variables partly explained the observed yield variation. Other biotic or abiotic factors that were not aggregated in the independent variables, such as competitions with native species that were incompatible with the introduced rhizobia, impaired the nodulation and affected yield. On the other hand, the rate of P applied was only 20 kg ha^−1^, which was insufficient to achieve optimal soybean yield. Future studies to address the “non-compatible hypothesis” and the optimal P rate for each agroecological zone will deserve further research investigations.

### 4.3 Soybean import reduction through the adoption of Rh inoculant and P fertilizers

Linking biophysical and economic models is important in a world facing the complexities of increasing crop production under pressing climate change threats (Islam et al., 2016). We conducted a two-scenario analysis of the Rh + P combination treatment, evaluated the possibility of adoptions that could take place in the future, and assessed the impacts on Nigerian food security and soybean trade. Results from the IMPACT model showed that the Rh + P combination has the potential to reduce the current soybean importation demand by a maximum and reverse the importation trend from 2029 if maximally adopted. The scenario analysis through the adoption of promising agricultural technology on yield by 2050 has been implemented in several commodities, including rainfed maize in Africa, irrigated rice in South Asia, rainfed potato in rainfed sorghum in India, and rainfed groundnut in Africa and Southeast Asia (Islam et al., 2016). In many of these studies, the authors observed that promising technologies tested in many regions/or ecologies showed a partial to complete offset of the deleterious impacts on yield through the adoption of technology (Islam et al., 2016). In the present study, we have opted for large adoption as many demonstrations, including many participating farmers, in various steps of soybean production and training in inoculation technology with the goal of rapid adoption, were implemented to increase soybean production and improve food security.

Possible gaps and limitations in the modeling for agricultural systems as presently conducted in this work are the bias generated from the model tools and environmental conditions such as soil and climate that are heterogeneous, especially in Sub-Saharan Africa. These gaps certainly decrease statistical robustness and bias upward the values obtained for decision variables, which could often be unachievable in the real world. To avoid these aggregated biases resulting from the model, the natural conditions of the independent variables were tested for data homogeneity. To minimize the biases from human heterogeneity at on-farms, we trained the engaged farmers to use Rh inoculant and P fertilizer technology, and VPs supervised their work regularly before trial establishments in the respective areas. Overall, the present results underscore the fact that innovative interventions should be tested across a wide range of AEZ, capturing all possible variables for wider adoption. Our results strongly suggest that the application of rhizobium inoculation is affordable, and represents a low-cost agricultural intensification strategy when combined with P fertilization and VPA technical assistance.

## 5 Conclusions

This study used a comprehensive mix-methodological approach integrating large-scale on-farm demonstrations and the engagement of local extension agents and farmers, as well as a machine learning approach, to identify the major determinants of yield variability in three savanna agroecological zones in Nigeria. The IMPACT model simulates the effect of the adoption of Rh + P on food security and imports to develop sustainable soybean production technology. Our result demonstrates a superior benefit from the combination of Rh inoculant and P fertilizer to improve soybean yield in the farmer field conditions of northern Nigeria. Soybean yield was well predicted from the combination of soil, climate, inputs, and crop management parameters in the northern Guinea Savanna agroecological zone, implying that the NGS offers a suitable production environment for soybean production among the three agroecological zones. If the combination of Rh inoculation and P fertilization demonstrated by this study as best practices and promoted by policymakers and maximally adopted by farmers in Nigeria, the country will reverse its dependency on soybean trade to about 21% by 2029 and become a self-sufficient producer by 2050 in the absence of climate change threats.

## Data availability statement

The raw data supporting the conclusions of this article will be made available by the authors, without undue reservation.

## Author contributions

Writing original draft: MJ. Methodology: MJ, KD, TE, TC, RM, MH, FS, and JJ. Manuscript review and inputs: MJ, KD, TE, TC, RM, MH, FS, and JJ. All authors contributed to the article and approved the submitted version.
