# Supplementary material for: Exploring the potential of mapped soil properties, rhizobium inoculation, and phosphorus supplementation for predicting soybean yield in the savanna areas of Nigeria
Source: Front Plant Sci. 2023 Apr 11;14:1120826. doi: 10.3389/fpls.2023.1120826 (PMC10126304; doi:10.3389/fpls.2023.1120826)
Supplement: Supplementary file 1 [file DataSheet_1.pdf]

Exploring the potential of mapped soil properties, rhizobium inoculation, and phosphorus supplementation for predicting soybean yield in the Savanna areas of Nigeria

Martin Jemo[1, *], Krishna Prasad Devkota[2]; Terence Epule Epule[3], Tarik Chfadi[3], Rkia Moutiq[4], Mohammed Hafidi[5], Francis B. T. Silatsa[1], and Jibrin Mohammed Jibrin[6]

Supplementary Table

Table S1. Skewness and Kurtosis test of the extracted soil properties used to predict yield variable from all sites, Sudan, northern Guinea and southern Guinea Savanna of Nigeria

	All sites		Sudan Savanna		Northern Guinea Savanna		Southern Guinea savanna	
	Skewness	Kurtosis	Skewness	Kurtosis	Skewness	Kurtosis	Skewness	Kurtosis
Effective Cation Exchange Capacity [cmol (+) kg $^{-1}$]	-0.07	-1.26	-0.12	-0.24	-0.42	-1.47	0.93	0.10
Exchangeable Ca [cmol (+) kg $^{-1}$]	0.71	-0.08	1.96	3.65	0.44	-1.08	0.18	-1.20
Fe content (mg kg $^{-1}$)	1.2	2.04	1.92	2.99	-0.01	-1.38	1.45	1.30
Exchangeable Mg [cmol (+) kg $^{-1}$]	0.68	-0.04	-0.22	0.42	0.39	-1.00	0.68	-0.24
Av-Pi content (mg P kg^{-1})	0.98	1.29	1.45	1.21	0.56	1.83	0.67	0.22
Exchangeable K [cmol (+) kg $^{-1}$]	-0.35	-0.88	-0.68	-0.35	0.10	-1.43	0.04	-1.43
Su content (mg kg $^{-1}$)	0.32	-0.56	0.14	-0.26	0.31	0.08	0.57	-1.02
Zn content (mg kg $^{-1}$)	0.67	-0.4	1.26	0.86	0.04	-1.50	1.03	-0.31
Organic carbon content (g kg $^{-1}$)	0.81	-0.53	0.36	0.49	0.10	-1.35	-0.26	-1.58
Total Nitrogen content (g kg $^{-1}$)	0.18	-0.94	1.04	1.94	-0.29	-1.37	0.44	-0.95
pH (H_2O)	-0.44	-0.37	-0.12	-0.24	0.10	-1.35	-0.26	-1.58
Clay content (%)	0.15	-0.65	0.35	-1.49	0.58	-0.62	-0.47	0.21
Silt content (%)	0.46	-0.53	1.04	0.01	0.40	0.00	-0.65	-0.81
Sand content (%)	0.26	-0.50	1.32	0.20	0.40	0.002	0.37	-0.86
